# The effect of attachment and environmental manipulations on cooperative behavior in the prisoner’s dilemma game

**DOI:** 10.1371/journal.pone.0205730

**Published:** 2018-11-12

**Authors:** Maliheh Taheri, Pia Rotshtein, Ulrik Beierholm

**Affiliations:** 1 School of Psychology, University of Birmingham, Edgbaston, Birmingham, United Kingdom; 2 Psychology Department, Durham University, Durham, United Kingdom; Universitat Jaume I, SPAIN

## Abstract

Cooperation and competition are vital for human survival and for social progress. In this study we examine the impact of external (environmental) and internal (individual differences) factors on the tendency to cooperate or compete in social conflicts. To this end, 53 young adults played blocks of the repeated Prisoner’s Dilemma Game with each other or with a computer. The environmental context was manipulated across blocks, by introducing uncertainty, randomly losing or gaining money. Individual differences were assessed by participants’ attachment style. We found that participants cooperated more when randomly losing money compared to when randomly winning or in the neutral condition. Moreover, in a negative uncertain environment, individuals with higher anxious and avoidant attachment styles cooperated less. The above effects were only observed when playing against a human and not a computer. Overall, the findings highlight the dependency of cooperative behavior on the context as driven by external and internal factors.

## Introduction

Cooperation and competition are vital for our survival in a complex social environment [1]. The tension between cooperative and competitive behavior is specifically emphasized in a conflict situation. An example is social dilemmas where the interests of the collective are in conflict with the individual’s self-interest [2]. In traditional economic theories decisions are assumed to reflect a ‘rational choice’, aimed to maximize individual gain [3]. However, many theories and empirical findings have challenged this simplistic view [4,5]. Specifically, in the context of social dilemma, cooperation is suggested to be driven by social factors [6–8], emotional context [9–12] and personality traits [13–15]. In this study, we specifically focus on the impact of the environmental uncertainty, and how individuals with different relational attachment style react to it in a repeated Prisoner’s Dilemma Game (PDG).

Economic games such as PDG, Ultimatum Game (UG) and Dictator Game (DG) measure individual’s responses in face of conflict between self-interest and collective interest. In the PDG, unlike some of the other games (UG, DG) both players have equal status and role. The players win or lose money based on their individual decisions to ‘cooperate’ or ‘defect’ (also referred to as ‘betray’ in the literature, we will use both terms interchangeably): if both players decide to cooperate they will be better off than if both decide to betray. Conflict arises when one player decides to cooperate while the other decides to betray, leading to an advantage for the betrayer and a cost for the cooperator. In other words, deciding to cooperate in the PDG means one must *trust* the opponent to cooperate as well, otherwise there is a risk to incur a large loss. Hence, PDG is used to assess cooperation in the context of trust.

Social contract theory originally proposed by Hobbes [16] suggests that cooperative behavior is one of the building blocks of societies. It postulates that despite an incentive to defect in some scenarios (e.g. PDG), it is socially agreed that for long term mutual benefit it is best to cooperate. Cooperation between humans is argued to be an evolutionary behavior that is crucial to our species survival [17]. Indeed, in the context of PDG (both in the single and repeated versions) cooperation is observed, although there is a large variability between studies in the extent of cooperative behavior [18]. Unsurprisingly, cooperative behavior in PDG is greater when playing against human partner as compared to when playing against computer partner [8], highlighting the social aspect of the game.

Cooperation has also been hypothesized to depend on factors external to participants. Economic hardship, food sparsity and limited foraging options has been anecdotally claimed to cause humans to be either more or less cooperative. For example, it is suggested that exposure to natural disasters leads to increased subsequent cooperation. Chantaral and colleagues [19] tested altruistic behaviors after a flooding disaster. Using a DG, the authors showed that those directly affected by the flooding were more generous compared to those who were not. There is also evidence that traumatic experiences in a community can lead to more cooperation [20]. Similarly, it has been shown that a positive national event, like winning an international football match, can lead to increased investments in national companies (increase in the FTSE 100 index when England wins [21]). Hence the external environment, or unexpected loss, may influence human cooperation at least on short time scales. This short-term-effects maybe mediated through changes in emotional states following the external events.

Over longer time scales, personality traits are likely to also affect cooperation. The ability to trust others in daily interaction can be affected by an individual’s attachment style. The attachment theory advocates that individuals interact with each other based on their internal relational “working model” developed in early childhood [22]. Attachment is categorized into secure and insecure types and is described along two dimensional axes: anxiety and avoidance [23]. Individuals with secure attachment score low on both the anxiety and avoidance axes. Secure attachment is associated with positive, constructive and trustful social interactions. On the other hand, individuals who have insecure attachment, tend not to trust others. Those with high score on the anxiety axis are afraid of rejection and seek closeness at all times. Individuals with high scores on the avoidance axis emphasize their independence and avoid forming relations.

Two studies investigated the impact of attachment on behavior in social dilemmas. Almakias and Weiss [24] examined how avoidance and anxious attachment styles affect decisions in the UG and DG. In contrast to the PDG, the relation between players in the UG and DG is asymmetric and assumed to measure sensitivity to fairness. In the UG one participant proposes a split of money, with a second participant holding a veto power, while in the DG the receiver has no say over the split. In the Almakias and Weiss study, participants played 40 trials of a one-shot game with different fictitious partners, half as proposers (DG) and half as receivers (UG). They reported that participants with anxious attachment style accepted smaller offers in the UG while also making higher offers in the DG. The authors concluded that this paradoxical behavior where anxious individuals always end up with less money than their opponents (either as proposer or receiver) suggest they have lower sensitivity to fairness. It is argued that the desire of a high relational anxious individual to be loved and appreciated leads them to reduce their own share and increases the other’s. On the other hand, participants with avoidance attachment style show the opposite pattern–they made smaller offers whilst they also rejected smaller offers (although this was not significant) suggesting that avoidance individuals have higher sensitivity to fairness, where they focus on protecting themselves from exploitation.

In contrast, in social conflict with symmetric relations and where trust rather than fairness is the focus, such as the PDG, individuals with anxious or avoidance attachment styles are reported to be less cooperative [25]. This was shown using 2 trials of a one-shot prisoner dilemma game (with two fictitious partners).

Attachment style not only affects the way people interact with others, but also affect the way individuals respond emotionally to various situations [26]. Individuals with high anxious styles show hyper emotional (positive and negative) response, while those with avoidant styles are associated with deactivation of positive emotions [27]. In support of this, empirical studies have shown that people with anxious relational attachment rated both positive and negative stimuli as more intense, independent of the social content; those with avoidant style rated positive social (but not non-social) images as less pleasant [28]. It is suggested that emotions activate the attachment internal working model [29].

Two opposing views have been proposed on the impact of attachment style on cooperation when in an uncertain/threatening environment. The affect reactivity and regulation model [30] posits that in an uncertain negative environment, individuals with high avoidance will distance themselves from others, and hence are likely to cooperate less; while individuals who have anxious relational style will seek more support, and hence potentially cooperate more. Alternatively, it has been argued that in uncertain situation, in which the behavior of the partner is unpredictable, individuals with insecure attachment (anxious and avoidance styles) expect the worst, showing overall less trust leading to less constructive cooperative behavior, even in repeated interactions [25].

The aim of the current study was to revisit cooperative behavior in the context of the social dilemma, examining the impact of attachment style as an internal factor and of the environment (rich (positive), neutral or scarce (negative)) as an external factor. We used the PDG as a manipulation of social dilemma because the relationship between the two partners is symmetric. Hence, we expected the PDG to specifically tap into trusting others’ behavior which is assumed to be impacted by the attachment style. To magnify the element of social trust in the game and its emotional and social connotation, we describe the two choices in the PDG as betray and cooperate. We used a repeated version of the PDG to allow reciprocal trust to be triggered across multiple encounters. The PDG task included a manipulation of the environmental context through unexpected monetary losses or wins. Thus, the PDG was played 1) in a positive context, where participants randomly received additional money, 2) a negative context, where money was randomly taken, and 3) in a neutral context where no money was won or lost randomly. This external variable emulated a period of safety/economic growth or threat/recession and would be expected to interact with the attachment style for cooperative decisions. Finally, to rule out that any effect observed is not specific to social interactions we manipulated the opponent; participants played against a real human (social) or a computer (non-social) partner. Subjects were aware who their opponent was when they played. It is important to note that in contrast to many studies using social dilemma games here participants interacted online with real humans whom they only briefly met at the beginning of the experiment.

While the role of attachment has previously been examined for PDG in single games [25] this is the first study to examine cooperation in repeated PDG where participants can build up trust over time, and the first study to examine how the cooperative effect of attachment style is further influenced by uncertain or threatening environments.

Based on literature on threats and uncertainty we expected participants to cooperate less when in a positive supportive context (randomly winning a sum of money) and more when in a negative threatening context (randomly losing a sum of money) and this effect is expected to be larger when playing against a human. How the internal factor (attachment styles) affects cooperation is less clear; previous studies reported that both anxious and avoidance relational styles trended toward less cooperation. We anticipated that external environmental factors (specifically threat and uncertain negative outcomes) would magnify the effects of the internal factors (the attachment style) on cooperative behavior; high avoidant attached participants showing a decrease in social cooperation in response to negative context. The impact of environment on the cooperative behavior of individual with anxious attachment style during interactions is less clear. Furthermore, we expected all of these effects to be specific to interactions with human participants, with generally lower cooperation with computer opponents.

## Methods and materials

### Participants and design

Sixty-two university students were recruited for the study. The criterion for inclusion was any adults age 18 and above. Participants were tested in pairs, based on opportunistic matching. Two participants who signed on to the same time slot played together, on the condition that they did not know each other in advance. A follow up lifespan study (not reported here) revealed that the impact of attachment style on cooperative behavior changes with age. Therefore, in the current study we excluded 9 participants whose age was above 30yr. This resulted in 53 participants, each of whom received £5 for their participation plus any additional money they won during one of the blocks played in the game (see below). Participants were fully informed about the task both in writing and verbally before giving their written consent. The study procedure was approved by the University of Birmingham Science, Technology, Engineering and Mathematics (STEM) Ethical committee.

The study design was a mixed design with partner (computer, human) and environmental context (positive, neutral, negative) as within-subject factors and attachment styles (anxious, avoidance) as between subject covariates.

### Task

**Fig 1:**
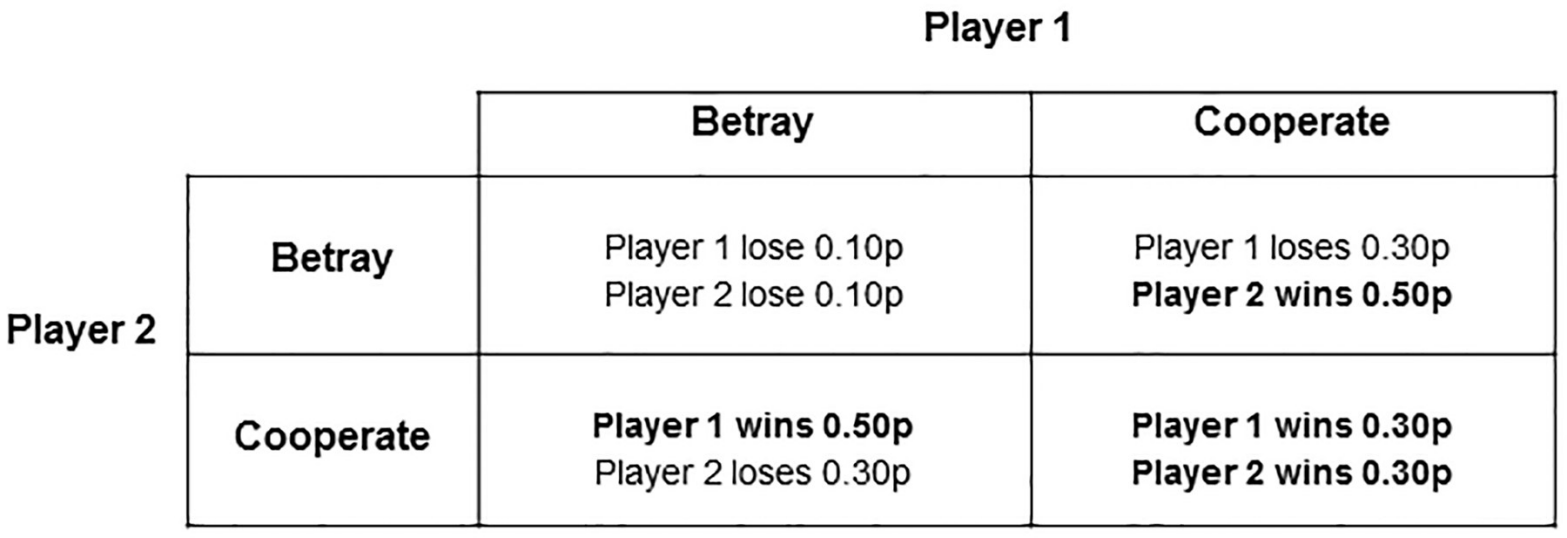
Payoff table. Each player had two options, cooperate or betray, and there are 4 outcomes based on both players’ decisions. The payoff scheme is designed to encourage betrayal, as betraying assuming the other cooperates is associated with the highest gains, as in the original PDG.

Participants played a modified version of the repeated prisoner’s dilemma game in pairs. The aim of this game was to win as much money as possible. The amount of money that could be won on each trial depended on both players’ decisions. On each trial participants had to choose whether to ‘cooperate’ or ‘betray’ (by pressing 1 or 2 on the keyboard). If both participants cooperated they won 30 pence (30p) each; if both betrayed they lost 10p each. If one betrayed whilst the other cooperated the one who betrayed won 50p and the one who cooperated lost 30p (Fig 1). The payoffs were designed to encourage betrayals as this was associated with the highest payoff assuming the other participant cooperated.

Participants played with either a real human partner (the other participant) or a computer partner in separate blocks. At the beginning of a block they were informed whether they were playing a human or a computer. The computer used a tit-for-tat strategy, collaborating in the first trial, then subsequently performing the same choice as the participant’s previous decision. However, to mask this strategy in 20% of the trials the computer made a random decision. The use of the tit-for-tat strategy was done to emulate as much as possible the conditions of playing with another human by emphasizing reciprocation. In addition, tit-for-tat is a well-known strategy that emphasizes initial cooperation, but which immediately responds to any deflection by immediate mutual deflection. The strategy has been used to explain aspects of human decision making and has the advantage of being simple yet effective [10,11,31].

The environmental context was manipulated across blocks. We used three contexts: negative, neutral and positive contexts. In the negative blocks, random monetary loses (20p) were introduced on 30% of the trials. In the positive blocks, random monetary wins (20p) were introduced on 30% of the trials.

Subjects played with each partner (human or computer) in all three environmental contexts, for a total of 93 trials divided into six experimental blocks of 13 to 17 trials. The number of trials per block varied between blocks to prevent the use of strategic decision making on the last trials. The order of the six blocks was counterbalanced across participants.

### Experimental procedure

Participants were tested two at a time with the condition that they did not know each other beforehand. At the beginning of the session, the instructions of the game were given to both the participants in the same room. They were introduced to the aim and structure of the game and the payoff table was clearly explained to them. The environmental and the partner manipulations were also explained, and subjects were advised that this would be fixed across blocks of trials. They were then taken to two separate rooms, where they would play the game. This precluded any communication between participants during the game.

In separate rooms, participants practiced the task and the experimenter verified that they understood it. The experimenter left the room before the actual game started, to ensure they would not bias decisions.

**Fig 2:**
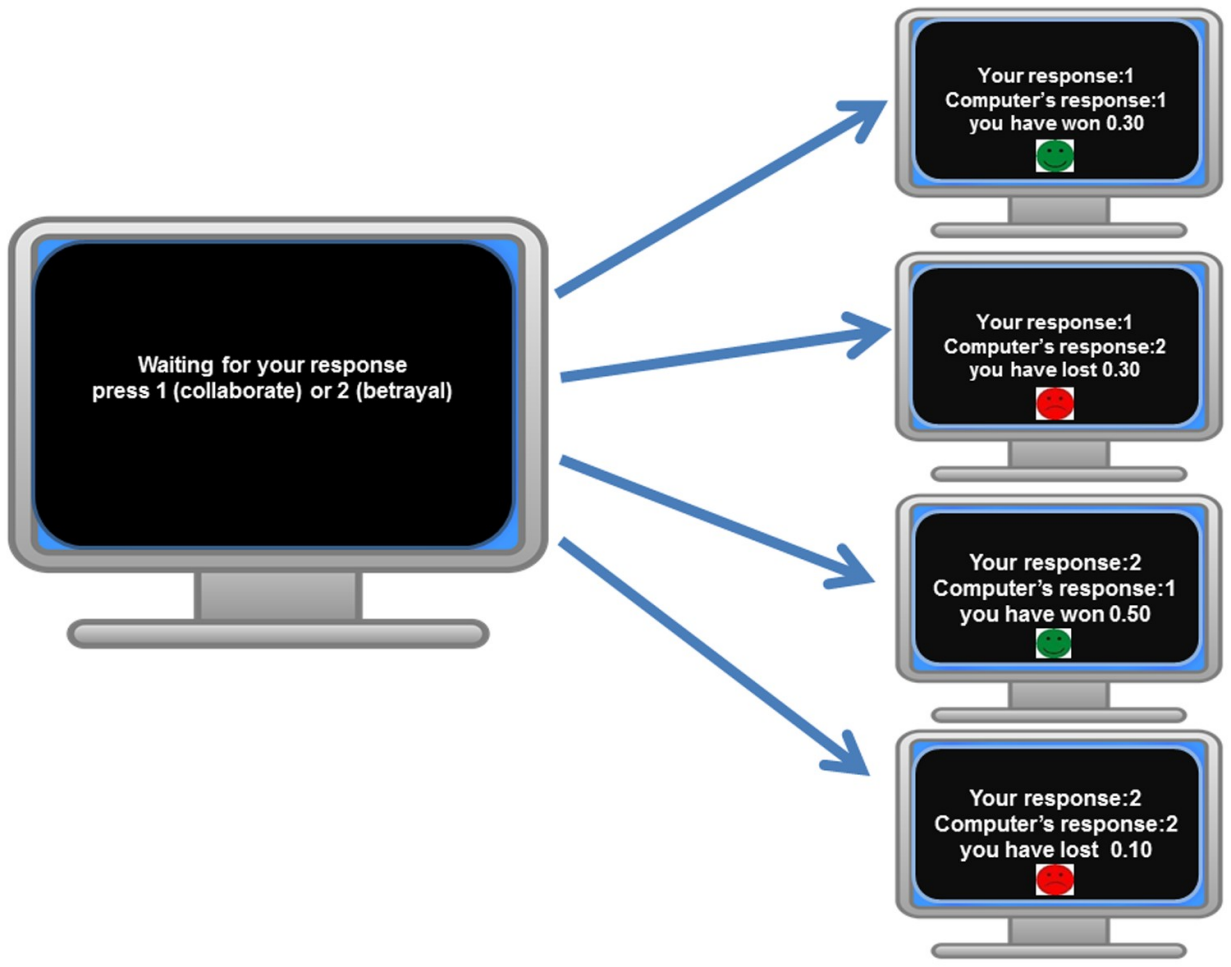
Choice and feedback screen. Participants were presented with two options and the choice screen remained until both participants made a response (pressing keys 1 or 2). Right side: Depending on the responses of both participants one of four potential feedback screens were presented (2 seconds). In the positive & negative environmental context there was a 30 percent chance of a screen informing subjects of a further random gain/loss, before the beginning of the next trial.

Each block started by informing each subject of the type of partner they would encounter (“In this block you will play against a computer (human) partner”). This stayed on the screen until the participant was ready to start. Participants where then presented with the response screen stating: “Waiting for your response, press 1 to cooperate or 2 to betray”. After both players decided, a feedback screen appeared notifying both participants of their decisions and the money won/lost for each (see Fig 2).

During the negative context block in 30% of the trials a screen with an unhappy red face appeared after the feedback screen, displaying the following message: “Oops you have accidently lost 0.2”. Similarly, during 30% of the trials under the positive context, after the feedback screen a smiling green face was displayed with the message: “Congratulations, you have won extra 0.2”. This context screen was displayed until the subject made a button response.

For the analysis, we measured cooperation ratio, the number of trials where the decision was to cooperate relative to the total number of trials in the block, and response time.

### Attachment self-report

On the completion of the game, in the individual rooms, participants were asked to fill out 5 personality and trait questionnaires (UPPS+, BISBAS, RAAS, ECR and the Big five). For the purpose of the current study we focused only on the questionnaires that assessed attachment style.

Attachment was assessed in the context of intimate relationship using the Revised Adult Attachment Scale (RAAS) [32]; and close relationship using the Experiences in Close Relationships Inventory (ECR) [33]. RAAS and ECR measure anxious and avoidance attachment styles in the context of adult relations. The RAAS was completed before the ECR. Example of questions assessing anxious style in the RAAS (6 Items) is: “I often worry that romantic partners won’t want to stay with me”, and in the ECR (18 Items): “My desire to be very close sometimes scares people away”. A question that assess avoidance style in the RAAS (12 Items) was: “I find it difficult to allow myself to depend on others” and in the ECR (18 Items): “I find it difficult to allow myself to depend on others”. In the RAAS participants responded using a scale of 1 (strongly disagree) to 7 (strongly agree); in the ECR they responded using a scale of 1 to 5 (disagree—to—agree, respectively). We used two questionnaires to assess attachment styles in adulthood in order to increase the reliability of the self-reported measures.

In the analysis, we separated responses to questions that measure anxious and avoidance styles. Given the relatively high overlap of the two questionnaires, the data was combined using principle component analysis (PCA). For each sub-scale, we used the first component, i.e. the one that explained the largest amount of the shared variability in these measures (see Results below). Thus, each participant had one score reflecting the level of relational anxiety and one score for relational avoidance attachment style. The attachment measures for relational anxiety combined of the RAAS and the ECR found high reliability (24 items; Cronbach’s alphas α = 0.85). The Cronbach’s alpha for relational avoidance of the combined attachment scores also found high reliability (29 items; α = 0.68).

## Results

### Attachment styles

The distribution of attachment style scores matched responses reported in the healthy population [26] (RAAS-anxiety: mean = 2.72, median = 2.83; RAAS avoidance: mean = 2.81, median = 2.75; ERC-anxiety mean = 3.41 and median = 3.29; ERC avoidance mean = 2.98 and median = 3.11).

The scores from the two questionnaires showed high and significant correlation for the RAAS and ECR relational anxiety sub scales, *r* = 0.79, *p* <0.001, as well as for the RAAS and ECR avoidance scores, *r* = 0.56, *p* <0.001. The scores from the relational anxiety and avoidance subscales on the other hand were near orthogonal, with no correlation between them on either questionnaire (RAAS: *r* = -0.025, *p*>.05; ERC *r* = 0.25, *p*>.05). This demonstrates that the two subscales assess different components of adulthood attachment styles.

**Fig 3:**
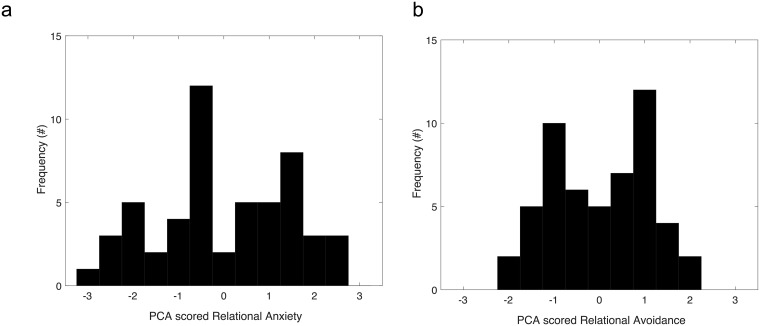
Relational attachment. Distribution of relational avoidance and anxious attachment styles in the shared PCA score. Low score means less secure attachment.

To achieve a more reliable measure for the relational styles, we used PCA to extract the shared component underlying the two questionnaires of each of the subscales. For relational anxiety the shared component explained 84% of the data and was loaded on both the RAAS and the ECR scales. For the relational avoidance style, the shared component explained 86% of the variability and was loaded on both the RAAS and the ERC scales. The combined score on each sub-scale was mean scaled before it was used as a covariate in the analysis. Fig 3 presents the distribution of individual scores on each relational attachment styles (after they were means scaled). The distribution of the combined relational avoidance score (on the right) was slightly positively skewed, while for the combined relational anxious score (the graph on the left), the distribution is approximately normal.

### Validation of the environmental manipulation

Participants played the game in three environmental contexts: negative, neutral and positive. To validate the impact of environmental manipulation we used two methods: 1) explicit questions: we asked a sub-sample of 26 participants to report their emotional responses for receiving random wins or losses. This was done after completing the experiment. Of this sub-sample 54% reported that they were not emotionally affected by the manipulation; and 35% reported positive feeling (e.g. joy, encouragement) after receiving a random win; while 23% reported negative feeling (e.g. annoyed, frustration) after receiving a negative loss; some participants were affected by both negative and positive environmental manipulation. Thus, the self-reported data suggested that while subjects were clearly aware of the manipulation only about half of the participants admitted or were consciously aware of being affected by the environmental manipulation.

The second method for validating the environmental manipulation was an implicit measure of response time (RT). Here it was assumed that a consistent change in RT in response to the environmental manipulation will reflect a change in participants’ internal states. The analysis excluded four participants that presumably initiated their response before the decision slide, showing average responses below 10ms; for the rest of the 49 participants, decision time took longer than 500ms.

**Fig 4:**
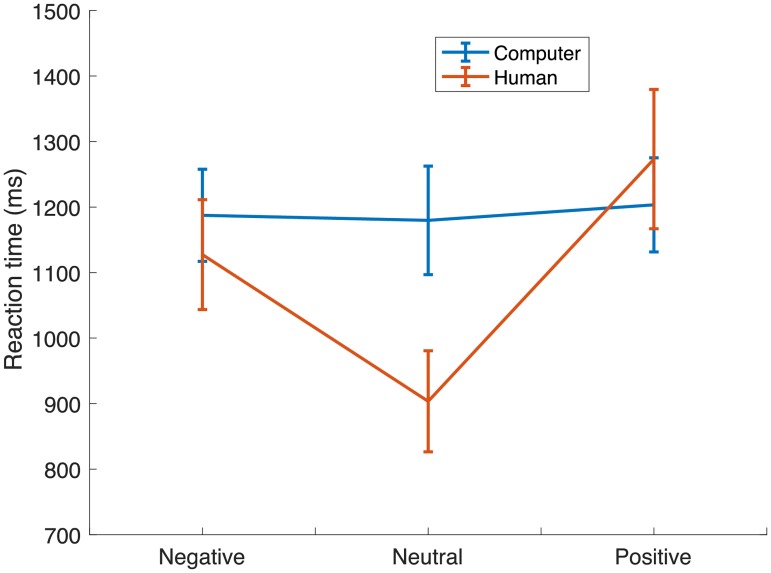
Response time when playing against a human and a computer. Response time when playing against a human and a computer partner across the three environmental contexts. Subjects were slower in the negative and positive environments when playing against a human partner. The error bars are standard errors.

The decision time data was used in a 2 (partner: computer, human) * 3 (context: negative, neutral, positive) ANCOVA test with attachment as covariate. The type of partner marginally affected response times to decide, *F* (1, 46) = 3.47, *p* = 0.08, pη^2^ = .065. More importantly there was a main effect of environmental context, *F* (2,92) = 5.76, *p*<0.01, pη^2^ = .11, Participants were fastest to make a decision during the neutral context blocks, *mean* = 1041.65ms, *std* = 72ms, and slowest during the positive, *mean* = 1238.31ms, *std* = 79ms, while decisions during the negative environmental blocks were in between, *mean* = 1157.43ms, *std* = 73ms. The effect of the environmental context depended on the type of partner, *F* (2,92) = 3.56, *p*<0.05, pη^2^ = 0.072. The interaction showed that the environmental context affected decision times only when playing with a human, where relative to the neutral context decision times were slower in the negative, *t* (48) = -3.2, *p* <.005, and the positive, *t* (48) = -3.2, *p* <.005, contexts. When playing with a computer, the environmental manipulation had no effect, t (48) = -.298, *p* = .7. This suggests that the environmental context primarily affected decisions in the social context (Fig 4).

### Effect of environmental context and relational styles on cooperation

**Fig 5:**
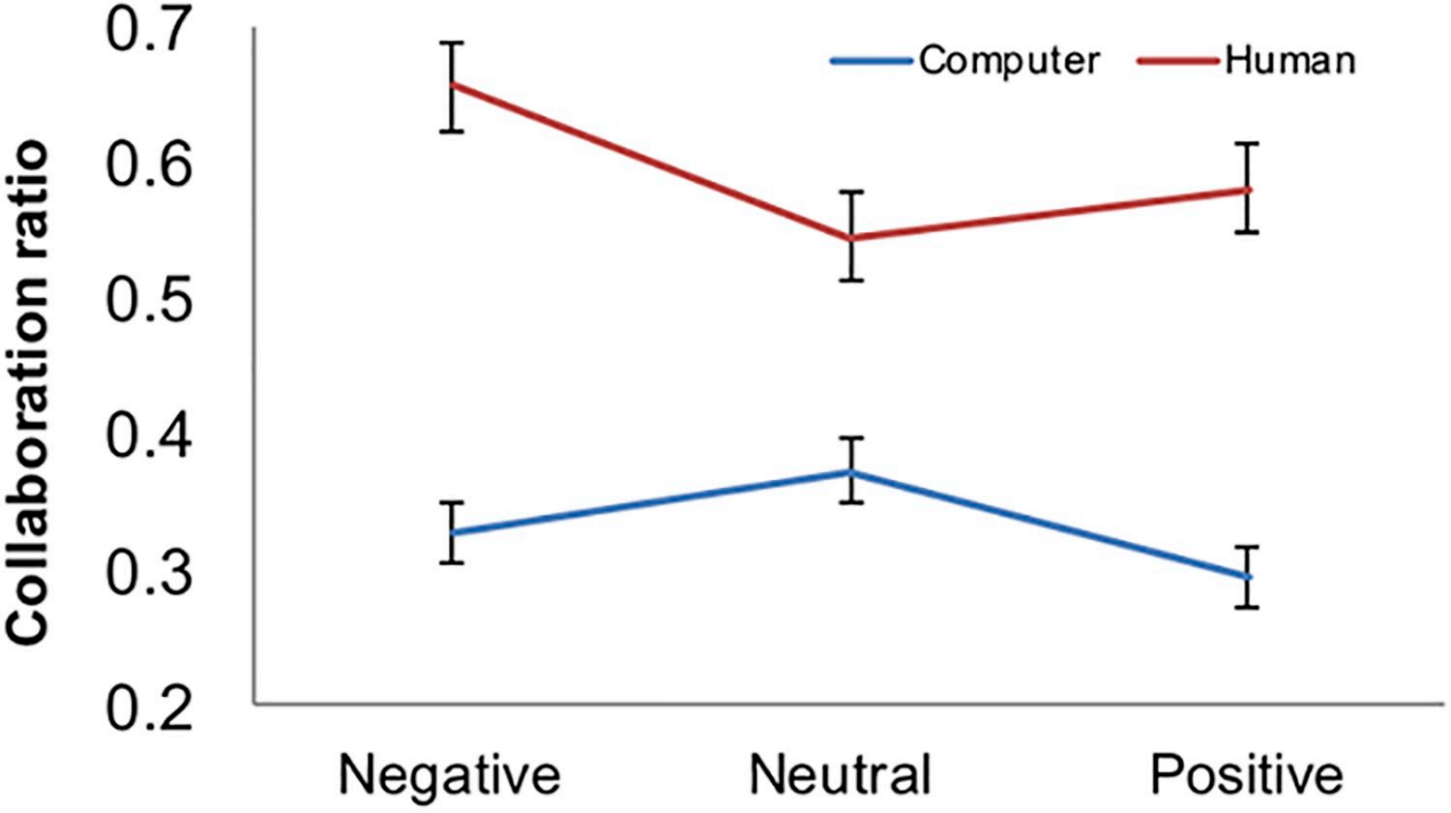
Cooperation with human and computer. Proportion of collaboration with human and computer partner across the three environmental contexts. Subjects collaborated more with human than computer partner and were overall less likely to cooperate in the positive environment (see main text). The error bars are standard errors.

Based on our hypotheses we focused on the proportion of times participants cooperated in each condition. We used an ANCOVA with partner and environment as repeated factors and attachment type as a covariate. The data showed a clear effect of partner, *F* (1, 50) = 28.56, *p* <.001, pη^2^ = 364, participants were more likely to cooperate with a human than with a computer partner (Fig 5). There was no significant main effect of the environmental context, *F* (2, 100) = 2.19, *p* >.05, pη^2^ = 0.041, but more interestingly a significant interaction between the environmental context and the type of partner, *F* (2, 100) = 4.93, *p* <.05, pη^2^ = .09. Further analysis showed that the interaction emerged because the environment only affected participants when playing with a human partner, *F* (2, 100) = 4.65, *p* <.05, pη^2^ = .085, but not when playing with a computer partner: there was more cooperation in the negative environment as compared to neutral, *t* (52) = 2.91, *p* <.05 and marginally more cooperation compared to positive environment, *t* (52) = 1.77, *p* = .08. This effect was not significant when playing against a computer (Fig 5).

Avoidance or anxious relational style did not reliably affect the overall proportion of cooperation, *F* (1, 50) = 3.25, *p* = .077, pη^2^ = .061; *F* (1, 50) = .11, *p* = .74, pη^2^ = .002, respectively. There was also no interaction between the type of partner and the relational style. However, avoidance relational style affected the response to the environmental context, *F* (2, 100) = 3.19, *p* <.05, pη^2^ = .060. There was also a three-way interaction of partner and environment with anxious, *F* (2, 100) = 4.37, *p* <.05, pη^2^ = .080 and a trend of three-way interactions of partner and environment with avoidance, *F* (2, 100) = 2.37, *p* = .098, pη^2^ = .045 relational styles. Post-hoc power analysis suggests that for the interaction effects observed in the data the power of the current study was higher than .70 for the relational anxiety and higher than .58 for the relational avoidance.

**Fig 6:**
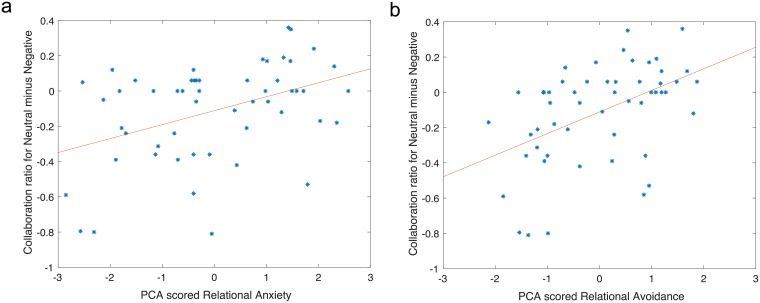
Correlation between shared PCA attachment scores and cooperation ratio. Differences in the cooperation ratio across negative and neutral environmental contexts correlated with shared PCA components for attachment relational score. High anxious individuals (on the left panel) tended to cooperate less when in a negative environmental context than when in a neutral environmental context. High avoidance individuals (on the right panel) were less likely to cooperate when in the negative context than in the neutral context.

Our a-priori hypothesis concerned the effects of attachment style on the pattern of cooperation when playing with human in a negative environment. Therefore, the follow up analyses focus on this question. We computed for each partner condition a differential environmental context score. This was done by subtracting the cooperation scores in negative and positive conditions from neutral condition. We then correlated these differential cooperation scores with the anxious and avoidance relational styles. To correct for the multiple comparisons, we used Bonferroni correction (corrected p value: .05/4 = .0125). For the anxiety relational style, the results showed that when playing with a human partner, high anxious individual tended to cooperate less when in a negative emotional state than when in a neutral emotional state, *r* (n = 53) = 0.41, *t* (52) = 3.24, *p* <.125, (Fig 6). Similarly, for the avoidance relational, when playing with a human partner high avoidance individual were less likely to cooperate when in the negative state than in the neutral state, *r* (n = 53) = 0.48, *t* (52) = 3.94, *p* <.0125. No significant result was found for the positive minus neutral contrast. When playing with a computer, cooperation ratio during the positive or negative versus the neutral states was not affected by anxiety or the avoidance relational styles.

In the current experiment, participants played with other participants in half the trials. This means that, like in real life, the behavior of any given participant depended to a degree on their partner’s and the relationship they formed in the game. In other words, the participants cannot be considered as fully independent samples, as their responses were dependent on the pairing. The lack of independence violates ANOVAs assumptions and may have led to inflation of the degrees of freedom. To ensure the pattern of results was not affected by this procedure, we ran two additional analyses: 1) A non-parametric analysis where independence between measures is not assumed; 2) An analysis where the averaged pair performance was the random variable.

The non-parametric analysis used Friedman’s two- way analysis of variance by ranks: with the 2 types of partners by 3 environment conditions. The model was significant (*p* <.0001) showing that the distributions of the data across the conditions were not the same. A follow up analysis showed that the average collaboration ratio when playing with a human was reliably higher than when playing with a computer (*p* <.001). Participant also collaborated more when playing with a human in the negative context than in the neutral context (*p* <.015). There was no reliable difference between negative and positive (*p* = .117) or neutral and positive (*p* = .451). Environment also affected the way participants played with the computer, showing less cooperation in the positive relative to the negative environment (*p* <.012), but no difference between the positive and negative (*p* = .202) and the negative and neutral environmental conditions (*p* = .423).

Finally, we used Spearman rho correlations to confirm the relations between cooperation ratio in the various conditions (neutral–negative; neutral–positive) and individuals’ reported attachment style. While at the group level, participants increased their cooperation when faced with adverse environment, this effect was diminished for high relational anxious (Spearman Rho = .356, *p* = .009) and for high relational avoidance (Spearman Rho = .499, *p* <.001) participants. Individual difference in attachment style did not affect the pattern of cooperation with computer or in the positive (vs. neutral) environment (*p*>.184).

In a second analysis we treated the pairs as the random factor, rather than the participants. The response of 22 pairs and their attachment scores were averaged (we excluded participants whose partner was excluded from the analysis due to age). ANCOVA with the factors partner and environment as repeated factors and attachment type as a covariate revealed a similar pattern. Participants were more likely to cooperate with a human than with a computer partner *F* (1, 19) = 12.52, *p* <.005, pη^2^ = .397, and the interaction of partner and environment affected the collaboration ratio, *F* (2, 38) = 3.66, *p* <.05, pη^2^ = .162. Follow up analysis showed that environment affected cooperation with human, *F* (2,38) = 3.33, *p* <.05, pη^2^ = .149, but not with computer partner.

The average attachment style of the pair also modulated cooperation dependent on the environment when playing with human but not with computer partner (*p*>.2). Specifically, when playing with a human, responses to the environment were affected by the averaged relational anxiety of the pair, *F* (2,38) = 3.672, *p* <.05, pη^2^ = .162; and the averaged relational avoidance, *F* (2,38) = 4.096, *p* <.05, pη^2^ = .177. Follow up analysis showed that the increased correlation during negative (vs. neutral), observed at the group level was diminished for pairs with high average of relational anxiety (r = .539, *p* = .01) and avoidance (r = .615, *p* = .002)

Taken together the data suggest clear effects of partner type and environment which were modulated by individuals’ relational attachment scores. These results were also observed using non-parametric statistical analysis, or when using reduced study power by considering the pairs rather than the individuals as the random factor.

## Discussion

In this study, we considered the effect of environmental context and attachment style on human cooperative decision-making in social and non-social contexts. We hypothesized that people make different decisions in a social conflict situation based on their attachment style and influenced by the environment. To test our hypothesis, we employed a modified version of the repeated prisoner’s dilemma game (PDG) and attachment questionnaires. Importantly, participants were tested two at a time to increase the validity of the social interaction and played multiple games with each other under different environmental contexts. We then compared individual differences in their cooperative behavior when playing with real human (the other participants) and computer partner under negative, neutral, and positive environmental contexts. Unsurprisingly we found that cooperation was higher when playing against a real human than a computer, but only, when playing with a real human did cooperation increase in the negative environment. High levels of relational anxious and avoidance styles were associated with tuning down the latter effect; reducing cooperative behavior in the negative context. We discuss these findings in the following sections.

### Effect of partner; playing with human or computer

Overall participants were more cooperative when playing with human partner as compared to computer partner. This finding is in line with previous research [6–8], and merely reflects the social aspect of the game. We observed that cooperation with human partner was higher, even though we used a repeated version of PDG (with a mean of 50 trials) that is shown to decrease cooperation [34].

It is worth noting that participants were matched to each other based on opportunistic sampling. Two participants signed up to each experimental time slots, and if they did not know each other they would proceed to play against each other. Participants were conscious that their decisions could affect the responses of the other, which potentially affected their tendency to cooperate. In our experiment, information gathered from post-experiment interviews of participants suggests that some were under the impression that humans in general are more cooperative and that they could influence their human partner’s decisions but not the computer’s. Hence, they believed that the computer was programmed to respond randomly (whilst the computer was programed to play a tit-for strategy in 80% of the trials, however participants were blind to this). Unsurprisingly, beliefs regarding the ability of one’s response to affect the response of the other player is important for cooperation.

A caveat of the above procedure is that differences in cooperation ratio between playing with a human or computer partner can be due to multiple reasons, which we cannot dissociate. For example, cooperation may be affected by 1) participants’ belief about the identity of their partner and the social acceptability of defecting; 2) their belief about the ability to modulate the behavior of their partner; and 3) while the computer played tit-for-tat with noise, real humans may not be as reciprocal. Thus the strategy of a computer opponent may have been qualitatively different than that of a human, despite the overall high performance of the tit-for-tat strategy [31].

### Effect of environmental contexts on cooperative responses

In this study, we manipulated the environmental context as an external factor, by introducing blocks with random monetary rewards or losses. Overall, in blocks when participants randomly lost money (negative context) they cooperated more as compared with the blocks where they randomly won money (positive context) or the neutral blocks. This effect was more reliable when playing against a human partner as compared to computer partner. In other words, participants betrayed more (potentially showing selfish behavior) in a rewarding or predictable environment compared with a threatening uncertain environment.

Human prosocial behavior often increases under negative circumstances. A good example of such altruistic and cooperative behavior can be seen in societies after natural disasters or wars [20]. Likewise, during periods of austerity less fortunate economic groups may be placed under extra negative emotional stress, which anecdotally may lead to more cooperative behavior [19].

From a cognitive perspective, the finding partially also supports the affect information processing idea [9,35]. This theory suggests that negative emotions (as elicited by threats and uncertainty) promote prosocial behavior, as individuals analytically analyze the details of the task and direct their attention to the external environment. Hence, participants are more likely to realize the long-term benefit of cooperation and consider the opponent and social norms when making decisions. However, the affect processing information also suggest that positive emotions leads to an optimistic view of outcomes, reliance on heuristics and attention to internal state. This often promotes less cooperative behavior as an individual may seek easy gain.

In the current study a positive supportive environment (winning random money) did not affect cooperative decisions. This is surprising, as the emotional validation measures (the analysis of the subjective reports and RT) showed larger impact of the positive (randomly winning money) than the negative (randomly losing money) environment. The literature on this aspect is less clear and it could be that in the context of trust games positive emotions do not affect pro-social decisions [11]. Thus further research is needed to clarify the impact of positive supportive environments on decision making during social dilemmas.

### Cooperation and rational attachment based on different environmental context

We found that relational style modulated the impact of random monetary loss (inducing negative mood) on cooperative behavior. While on average participants tended to cooperate more with their human partner during monetary loss blocks, those with higher relational anxious or avoidance scores were less likely to cooperate in the negative context as compared to the neutral environmental context when in the social context and playing with a human partner. In other words, relationally anxious and avoidance participants were found to react to this negative environmental context by behaving less cooperatively with their human partner. These finding accords with a report that individuals with insecure attachment tend to trust less initially, and following repeated interactions [36].

The observation that individuals with high avoidance relational style make less pro-social behavior accords with previous observations [24,36]. McClure and colleagues (2013) show that individual with high relational avoidance tended to cooperate less in the PDG. They reported that individuals with high relational avoidance tended to show more selfish behavior in the UG (less generous offer) and were less likely to accept offers in the DG. In both these previous studies the effects were weak and unreliable. One important difference between these previous observations and the current study is the introduction of the environmental manipulation. Indeed, we observed the impact of relational avoidance on decision only in the negative environment. As emotions linked with the environment are assumed to trigger the attachment style’s working model [24] it is likely that manipulation increased the impact of attachment on behavior. It is possible that relational avoidant participants’ decision to cooperate less is driven by fear of betrayal which increases in a negative environmental context. This could also be an indication of their difficulties in regulating their negative emotions and its impact on their interpersonal strategies as a result [30].

Like high relational avoidance, we observed that high relational anxious participants also cooperate less. This finding accords with the observation of McClure and colleagues [25], showing less cooperation in PDG. As above, McClure at al. reported the effects of relational anxiety on decision were weak and unreliable. Though here, through the introduction of the environmental manipulation the effects were larger and more robust. The current finding may appear to contradict those reported by Mikulincer et al. [27]. The latter report that relational anxious behaved in an opposite way to the relational avoidance participants, making more generous offers (UG) and accepting lower offers (DG). It is possible that differences in the relationship structure between the two players in these games can explain the apparent discrepancy of the findings. As mentioned in the introduction, the ultimatum and the dictator games are based on asymmetric power relations, where resources are held by one of the participants and responses are made based on known (DG), or partly predictable (UG) behavior of the other. While in the prisoner’s dilemma game, there is symmetry in power, the behavior of the other is less predictable, and trust between equals determines the distribution of the rewards. Thus, it could be that relational anxious individuals, being less trustful especially in a negative context are less likely to cooperate when the behavior of the other is less predictable. Another important contextual difference is the possibility to lose money in the PDG, while in the UG and DG one cannot lose money, but just not gain a reward. Thus, the impact of loss aversion on interpersonal trust is likely to be larger in the PDG. In PDG, if an anxious attached participant decides to cooperate whilst the other participant betray, the cooperative player loses more money (30p in our game), and if they both betray, they both lose smaller amount (10p in our game). The potential of loss is higher for a cooperating decision. However, in the ultimatum game if an anxious participant offers bigger amount and gets rejected (that is less likely) by the other player, they both get nothing, and if they reject smaller offer, they also get nothing. The ultimatum game thus does not allow for monetary loss (just lack of rewards), which might be more frightening or upsetting. As above, the inclusion of the mood manipulation is likely to have affected the result. The high sensitivity to the negative signals (random loss of money) by anxious attached individuals triggered their attachment system and therefore resulted in untrusting behavior due to fear of betrayal [24].

Taken together, we suggest that people with relational anxiety or avoidance perceived random loses in the negative environmental context as threat signals, significantly affecting their behavior; this was particularly seen in the social context, meaning when they were playing with their human partner and not with the computer partner. We propose that negative environmental context triggers the attachment system in both anxious and avoidant individuals and results in defensive behaviors, such as betrayal of their human partner.

Less cooperative behavior in negative environmental context in both anxious and avoidant relational style could be also due to an individual’s internal working models., as suggested by Bowlby [23]. Based on his theory an internal working model affects how we perceive and react to the environmental cues. This can be also explained by Bartholomew’s four dimensions model of attachment, according to which people who have a negative model of self and negative model of others are in the high anxiety axis [37]. According to Mikulincer, Shaver and Pereg people with high attachment anxiety have difficulties in regulating their negative mood and impulses [27].

### Limitations of our study and suggestions for future research

One limitation of our study, shared with most versions of the PDG, was the un-natural conditions of the experiment. Whilst in our study we tried to simulate the conditions that people have to make a decision in a conflicting situation with the minimum knowledge of their unknown partners, this was however done over computers and within experimental cubicles. While social interactions involving trust in a computer-based environment are now quite common, it would nevertheless be interesting to study human decision-making in a more natural and social environment. We do note though that, in contrast to many other studies, participants in this study met their opponent at the briefing session, highlighting the social context of the study while potentially allowing for less precise control over experimental conditions.

To further encourage social cooperation, we explicitly referred to the two choice options for the subject as ‘cooperate’ and ‘betray’. This is in contrast to the way the task is often presented [13] where the two options are merely referred to as ‘A’ and ‘B’ (e.g.) without any specific connotations. In our study, we wanted to explicitly engage attachment related systems of participant, hence these terms were beneficial.

The environmental manipulation, done by providing a random monetary gain or loss, may not necessarily have affected our participants. Though only slightly more than 50% reported being explicitly affected by the manipulation, average RT showed an effect of the manipulation. It could also be that the environmental manipulation and the experimental outcome confounded each other, as both were associated with monetary wins and losses. For example, the impact of random loss on mood may have been magnified following a loss during the trial, or lessened following a gain in the trial.

As participants played iterative games (~18 games x 3 blocks) with each other, we often observed that results tended to converge on either Nash equilibria. In other words, in many blocks participants’ responses were of mutual cooperation or mutual defection, hence there was relatively little variability and exploration in these blocks. This obviously hinders the ability, and reduces the power, of the study to test the impact of external and internal factors on cooperative behavior, due to ceiling or floor effects.

Lastly, it is hard to isolate the behavior of one participant in an iterated prisoner’s dilemma game as the behavior of one player affects the other player’s behavior, and vice versa. This leads to a unique pattern between the two players. Hence the interactions between two players are never random, but exhibit some kind of heterogeneity and potentially depend on the formed relation and impression made initially and through the game. For example, it is likely that an individual will change their cooperation tendency as a function of their opponent in a game, irrespective of their relational attachment style. Therefore, across participants the games were not fully equated, adding unaccounted variability to the measurement. However, the only way to void this is issue is to artificially control the opponent player (e.g. as hidden computer player), which will lessen the ecological validity.

### Conclusions

The current study showed that both environmental context and its interaction with attachment style affect our cooperative behavior in a social conflicting situation such as PDG. Specifically, the data suggests that whilst a negative environmental context facilitates human cooperation in general, it has a negative impact on both avoidance and anxious attached individuals and results in their decreased cooperative behavior. These results highlight the interaction between environment and attachment as important factors for individual decision-making.

## Supporting information

S1 File Data from 53 participants. (XLSX)
